# China’s pathway to a low carbon economy

**DOI:** 10.1186/s13021-019-0130-z

**Published:** 2019-11-21

**Authors:** Wenjuan Yang, Rongqin Zhao, Xiaowei Chuai, Liangang Xiao, Lianhai Cao, Zhanping Zhang, Qinglin Yang, Lunguang Yao

**Affiliations:** 1 School of Surveying and Geo-informatics, North China University of Water Resources and Electric Power, Zhengzhou, 450046 Henan China; 2 School of Geography and Ocean Science, Nanjing University, Nanjing, 210023 Jiangsu China; 3 Collaborative Innovation Center of Water Security for Water Source Region of Mid-Route Project of South-To-North Water Diversion of Henan Province, Nanyang Normal University, Nanyang, 473061 Henan China

**Keywords:** China, Pathway, Low carbon, Economy

## Abstract

Climate change has emerged as one of the most important environmental issues worldwide. As the world’s biggest developing country, China is participating in combating climate change by promoting a low carbon economy within the context of global warming. This paper summarizes the pathways of China’s low carbon economy including the aspects of energy, industry, low carbon cities, circular economy and low carbon technology, afforestation and carbon sink, the carbon emission trading market and carbon emission reduction targets. There are many achievements in the implementation of low carbon policies. For example, carbon emission intensity has been reduced drastically along with the optimizing of energy and industry structure and a nationwide carbon trading market for electricity industry has been established. However, some problems remain, such as the weakness of public participation, the ineffectiveness of unified policies for certain regions and the absence of long-term planning for low carbon cities development. Therefore, we propose some policy recommendations for the future low carbon economy development in China. Firstly, comprehensive and long-term planning should be involved in all the low carbon economy pathways. Secondly, to coordinate the relationship between central and local governments and narrow the gap between poor and rich regions, different strategies of carbon emission performance assessment should be applied for different regions. Thirdly, enterprises should cooperate with scientific research institutions to explored low carbon technologies. Finally, relevant institutions should be regulated to realize comprehensive low carbon transition through reasonable and feasible low carbon pathways in China. These policy recommendations will provide new perspectives for China’s future low carbon economy development and guide practices for combating climate change.

## Background

Implementing a low carbon economy has become a common concern in world scientific, political and public circles in the context of climate change and global warming. The aim of the low carbon economy is primarily to curb greenhouse gas emissions triggered by anthropogenic activities and mitigate climate change [1]. Recently, great efforts were devoted to the world’s transition to a low carbon economy. On the one hand, a series of policies have been issued to supervise the implementation of low carbon economy. For example, the United Nations Framework Convention on Climate Change (UNFCCC) was initialized in 1992 to accelerate the reduction of carbon emissions. Annual climate change conferences were held and many related agreements and policies have been formulated under the UNFCCC framework [2, 3]. In particular, in the Paris Climate Agreement, all Organization for Economic Co-operation and Development (OECD) countries pledged to curb carbon dioxide concentrations in the atmosphere at 450 ppm in an attempt to limit the global mean temperature to 2 °C [4]. On the other hand, many signatories have grappled with the best ways to reduce greenhouse gas (GHG) emissions [5] and officially pledged to control the amount of their domestic carbon emissions to a certain level [6]. For instance, European countries such as the UK, France and Germany set long-term targets to reduce carbon emissions by 60%, 75% and 80% in 2050, respectively, compared with the 1990 level [7]. By summing all such targets in the world, some scholars have suggested that the GHG emissions will be reduced by 80% between 1990 and 2050 [8]. Furthermore, ingenious and practical actions for a low carbon economy have been employed in many developed countries. These actions are principally focused on renewable energy [9], clean energy technology [7], carbon market and low carbon cities. For instance, the European Union’s Emissions Trading Scheme (EU-ETS), the first large-scale international market-based carbon emissions trading program, came into force in January 2005, and has since dominated the world carbon emission trading market. The Clean Development Mechanism (CDM) was successfully developed in the European Union, and has become one of the most important mechanisms of EU-ETS. In order to reduce carbon emissions and realize zero-carbon cities, the Greenest City Action Plan 2020 was formulated by Vancouver’s government, which has greatly promoted the development of low carbon cities. The above practices and actions provide important references for developing low carbon economy in developing countries.

Many scholars have examined the pathways of the low-carbon economy, including the aspects that are highly linked to the low carbon economy, such as resources [10], industry [11], electricity [12], buildings [13], cities [14–16], transportation [17] and low carbon technologies [18]. Moreover, a range of methods such as contrastive analysis [19], cluster analysis [11], scenario analysis [20], modeling approaches [21–23] and evaluation methodologies [24] have been frequently employed in low carbon economy studies. These studies have enriched our understanding of low carbon economy development; however, they mainly focused on the specific low carbon economy pathways. To our knowledge, studies on multiple pathways to a low carbon economy is absent at the country level. Therefore, this paper summarizes multiple pathways to a low carbon economy since 2000 to show how the low carbon economy has developed in China.

**Fig. 1:**
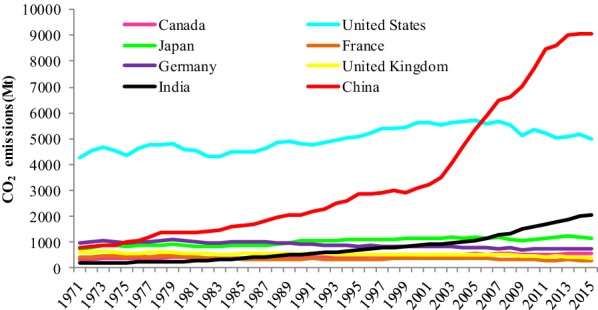
CO_2_ emissions of eight main countries between 1971 and 2015 (the main countries are eight historical or continual big emitters in the world. Different color lines represent different countries’ total CO_2_ emissions from fuel combustion between 1971 and 2015) Sources: IEA 2017 [52]

China has become one of the largest emitters in the world via the pathway of ‘high energy consumption, high GHG emissions’ since the reform and opening in 1978 [6]. Especially between 2000 and 2015, total amount of CO_2_ of China were 101,840.9 Mt, roughly 19 times those of France. Moreover, China’s CO_2_ emissions have increased sharply, compared with its own historical emissions. China’s CO_2_ emissions in 2015 were 10.6 times those of 1971, and China became the biggest carbon emitters in 2006 (1.6) (Fig. 1). As a large emitter, China is devoted to solving the environmental problems related to global climate change by developing a low carbon economy. President Xi Jinping proposed an ambitious blueprint of ecological civilization in the 19th National Congress of the Communist Party of China [25]. Recently, China has promised to reduce its carbon emission intensity to 60–65% of 2005 levels and achieve peak carbon emissions by approximately 2030 [26]. All of the efforts are aimed at low carbon economy transition of the whole China and making China a leader in global climate governance.

There are several problems in China’s low carbon economy pathways although these pathways have greatly contributed to GHG emissions reduction. Therefore, it is necessary to summarize low carbon pathways and describe their existing problems so as to provide experiences for future development. Based on the summarization of China’s policies and actions in combating climate change, this paper integrates China’s pathways to a low carbon economy from the perspectives of energy, industry, low carbon cities, circular economy and low carbon technology, carbon emission trading market, afforestation and carbon sinks, and carbon emissions targets. This paper also systematically discusses the achievements and existing problems in China’s pathways to a low carbon economy. Policy recommendations are also put forward to help provide new ideas for future low carbon development and guide future practices in combating climate change.

## China’s pathway to a low carbon economy since 2000

**Fig. 2:**
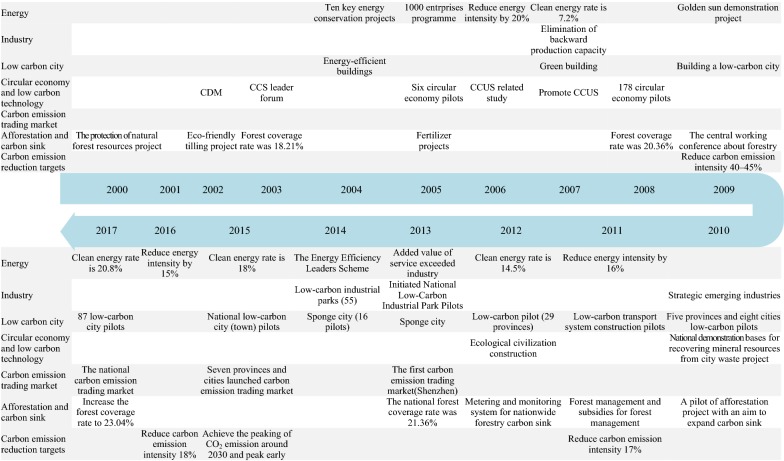
China’s pathways to a low carbon economy between 2000 and 2017 (the pathways contain eight aspects (energy, industry, low carbon city, circular economy and low carbon technology, carbon emission trading market, afforestation and carbon sink, carbon emission reduction targets) of China’s development in low carbon economy since 2000)

To develop the low carbon economy, China launched energy saving and carbon emission reduction targets in the 11th 5-year plan (FYP) in 2006 for the first time and promoted a series of actions and polices [27]. These actions and policies were mainly concentrated in the aspects of energy structure adjustment, industrial optimization, low carbon cities, circular economy and low carbon technology, carbon emission trading markets, afforestation and carbon sink projects. Many achievements have been made in the above aspects, which have greatly contributed to the low carbon economy development of China. Therefore, we focused on summarizing the pathways and illustrating the progress in the above fields (Fig. 2).

## Adjustment of energy structure

Energy consumption is intertwined with economy prosperity [28]. However, as the largest developing country in the world, China inevitably consumes a large amount of energy and causes GHG emissions. Therefore, curbing carbon emissions through energy conservation is vital for China. To encourage energy conservation nationwide, China revised the Energy Conservation Law in 2005 [29]. In order to improve energy efficiency in heavy industries, the Chinese government implemented the Ten Key Energy Conservation Projects [30] and the 1000 Enterprises Program [31] in 2004 and 2005, respectively. The above projects reduced energy consumption of 132 Mt standard coal equivalents by 2009 [32] and effectively boosted energy saving [33].

The Chinese government not only made efforts to curb energy consumption, but also attempted to adjust the energy structure. To achieve this, the Chinese government revised the Renewable Energy Law in 2005. In 2009, the government implemented the Golden Sun Demonstration Project [34] to increase the area of solar thermal application. The proportion of non-fossil fuel energy to fossil fuel energy was low in the 12s FYP (2011–2015) and rapidly increased to 20.8% by 2017 [35] because of the development of non-fossil energy such as hydro power, nuclear power, wind power, solar and other kinds of clean energy. Over the period 2000–2017, the rate of coal consumption reduced from 68.5 to 60.0% [36] by virtue of energy structure adjustment.

## Industrial optimization and upgrading

The prosperity of China’s economy is largely based on industries, especially those energy-intense industries that cause a large proportion of China’s total carbon emissions [37]. As a developing country, however, China has inevitably developed its economy to enhance comprehensive national strength. Therefore, it is vital to adjust the industrial structure along with economic development. In 2007, the Chinese government identified six energy-intensive industries for carrying out the Elimination of Backward Production Capacity project, which improves energy efficiency by replacing small thermal power generating units with larger ones [35]. Additionally, the government made efforts and provided support to vigorously cultivate and develop strategic emerging industries simultaneously since 2010, which also contribute to optimize industrial structure. In October 2013, Ministry of Industry and Information Technology (MIIT) and the National Development and Reform Commission (NDRC) started piloting low carbon industrial parks and established an evaluation index to examine the result of conducting the parks.

## Low carbon cities

Urbanization has predominantly resulted in energy consumption and carbon emissions. The proportion of global CO_2_ emissions related to cities is 70% [38]. The Average Annual Growth Rate (AAGR) of energy consumption was larger than the urbanization rate in China over the period of 2000–2017. Moreover, 16 Chinese cities were listed among the world’s 20 most polluted cities in 2004, and this alarmed policy-makers [39]. Hence, Chinese central government advocated low carbon cities construction and issued a series of pilots and policies for improving infrastructure in the current cities. Many cities issued their low carbon economy development implementation plans for low carbon cities construction after 2009. In sum, low carbon cities are mainly carried by low-carbon pilots, sponge cities, low-carbon community [40] pilots and national low-carbon city (town) pilots.

In 2010, the NDRC selected five provinces and eight cities to carry out low carbon pilots projects firstly [34]. Subsequently, the 29 and 45 provinces and cities low carbon pilots were carried out in 2012 and 2017 [41, 42], respectively. The number of low-carbon pilots reached 87 by 2017. Sponge cities were proposed by President Xi Jinping in 2013 and launched officially in 2014 [43]. In 2015, a total of 15 sponge city pilots were promoted. In 2013, the government began to carry out research on low carbon community pilots develop theory. In 2015, the NDRC issued the Notice on Accelerating National Low-Carbon City (Town) Pilots and eight national pilots were selected in the first batch [44]. The NDRC organized all of the eight low carbon city or town pilots to study and draft pilot implementation plans, guiding them to explore their own development modes featuring regional characteristics. The aims of these pilots were to focus on the integration of industrial development and urban construction, rational space distribution, intensive and comprehensive use of resources, low carbon and environmental-friendly infrastructure, low carbon and high-efficiency production, and creating a low carbon and comfortable lifestyle.

## Circular economy and low carbon technology

A circular economy is a crucial part of low carbon economy [45]. This section mainly discusses pathways of the CDM, Circular Economy Pilots, and carbon capture, use and storage (CCUS) in China.

The CDM was designed with two objectives: to contribute to local sustainable development in the host country and to assist Annex-I countries to achieve their emission reduction targets in a cost-efficient manner [46]. The CDM was introduced into China in 2002 firstly. Chinese government approved 4540 CDM projects which were focused on new energy, renewable energy, energy conservation, methane recycling et al. by August 2012. The estimated annual Certified Emissions Reduction (CER) of the approved CDM projects has reached 730 Mt of CO_2_e in 2012 [47].

The first batch of Circular Economy Pilots was launched in 2005. Since then, Chinese government stressed the importance of circular economy laws highly. Therefore, the Circular Economy Law was issued in 2008. By 2009, there were total 178 Circular Economy Pilots in China. In 2010, the National Demonstration Bases for Recovering Mineral Resources from City Waste project was launched.

CCUS has been acknowledged as one of the key low carbon technologies [48]. Moreover, in China, great efforts have been devoted to CCUS after Chinese leaders attended the Forum of Leader Activity in 2003. In 2005, the Ministry of Science and Technology (MST) of China and the European Commission signed a Memorandum of Understanding on CCUS to achieve Near-Zero Emissions Cooperation on coal use. This cooperation will be carried out in three phases. First, carrying out a pre-feasibility study on capacity building and demonstration projects; second, carrying out a feasibility study on demonstration projects; and third, building and operating CCUS demonstration projects in China.

## Carbon emission trading market

As an important financial policy, the carbon emission trading market has developed rapidly to curb carbon emissions in China in recent years [49, 50]. China launched carbon emission trading pilot projects in 2013 and established a national cap-and-trade system in 2017 to promote carbon emission reduction in enterprises. During 2008–2010, the NDRC tentatively selected specific districts to carry out exploratory rather than official carbon emission trading market. This exploratory exercise was preparatory for constructing a real carbon market. Based on this exploration, the NDRC selected seven provinces and cities to initiate carbon emission trading pilots in 2011 [47]. In June 2013, the first carbon emission trading market was launched in Shenzhen. Subsequently seven pilot carbon emission trading markets were launched in 2015 [44]. Excitingly, a national carbon emission trading market (electricity) was established by December 2017 [48]. The carbon cap-and-trade system has greatly contributed to the carbon emission reduction of enterprises in China.

## Afforestation and carbon sinks

The issues Reduced Emissions from Deforestation and Forest Degradation (REDD) has become a key topic of negotiations under UNFCCC [51]. Under the background of REDD, China vigorously promoted afforestation and forest conservation action to increase its carbon sinks. Consequently, the forestry carbon sink capacity was strengthened especially by afforestation in recent years. And according to a national forest resource investigation, the forest coverage rate was increased also primarily by afforestation from 1993 to 2013. For example, China launched the Key Shelterbelt Construction Project in Northeast, Northwest and Northern China in 1979. In 2000, the Protection of Natural Forest Resources project was issued in response to the severe reduction of natural forest during 1993–1998. The National Key Afforestation Projects, Key Shelterbelt Construction Project in Northeast, Northwest and Northern China (1979), Returning Farmland to Forests (1999), the Protection of Natural Forests Resources (2010), the Control over the Sources of Sandstorms Affecting Beijing and Tianjin (2002), and Key Shelterbelt Construction Projects in Yangtze River (1989) were also launched to enhance the carbon sink capacity of China. To further strengthen forest sink capacities, China launched the Pilot Afforestation Project with An Aim to Expand the Carbon Sink (2010) and Forest Management (2011). The National Data Base and Parameter Model Base for Forestry Sink Calculation and Basic Database of the Forestry Carbon Sink were completed in 2013 and 2015, respectively, becoming an important basis for scientifically calculating the forestry carbon sink.

The Chinese agricultural sector’s contribution to GDP is higher than that of many developed countries. Therefore, agricultural carbon emissions should not be overlooked in China. The Ministry of Agriculture (MOA) carried out Eco-friendly Tilling project in 2002 to reduce GHG emissions from the agriculture sector. This farming methods helps to improve the carbon storage capacity of farmland soil by 20% and cut farmland emissions of carbon and other greenhouse gases by 0.61–1.27 tons per hectare annually. In 2005, fertilizer projects were launched to guide farmers’ scientific fertilization nationwide. In 2015, the MOA launched the Campaign to Achieve Zero Growth of the Use of Chemical Fertilizer by 2020 and the Campaign to Achieve Zero Growth of the Pesticide Use by 2020 for increasing the formula fertilizers use efficient through cooperation between farmers and fertilizer production firms.

## Carbon emission reduction targets

During 2000–2015, the AAGR of China’s carbon emissions was 7.43%, nearly two times that of 1971–2000. Moreover, China’s annual carbon emission exceeded that of the United States in 2010 [52]. Consequently, China’s government has attached great importance to reducing the carbon emission intensity despite the amount of carbon emissions has increased rapidly over this period.

During the 11s FYP (2006–2010), China’s carbon emission intensity decreased rapidly although the total carbon emissions were increased. It was mainly because Chinese government made efforts by issued actions and policies to curb GHG emissions. For instance, the government stated that China would reduce 40–45% of its carbon emission intensity by 2020, compared with 2005 levels at the Copenhagen Climate Change Conference in 2009. Elimination of backward production capacity accelerated the progress of replacing small thermal power generating units with larger ones; strategic emerging industries were well developed by the end of the 11th FYP. The amount of non-fossil energy consumption and carbon emission intensity were constrained in 2011 for the first time in the 12th FYP. As a result, the amount of total carbon emissions increased slowly, and carbon emission intensity decreased rapidly over the period of 12th FYP. A carbon intensity reduction index has been included in the comprehensive evaluation of regional economic and social development in 2011. In the 13th FYP, in order to curb carbon emissions, the Chinese government stated that they will reduce energy intensity by 15% and increase the proportion of non-fossil energy to total energy consumption to 15% of the 2015 level.

## Achievements and existing problems of China’s low carbon economy

With the implementation of the above policies, great achievements have been made in the development of a low carbon economy in China. However, some problems remain that are associated with China’s current low carbon pathways, which should be avoided in future development.

## Achievements of China’s low carbon economy

Since 2000, China has adopted a series of measures to curb carbon emissions, including setting carbon emission reduction goals, elimination of backward production capacity, increasing carbon sink, low carbon city development, and conserving energy and improving energy efficiency. These measures have benefits for GHG emissions reduction and mitigating climate change.

**Fig. 3:**
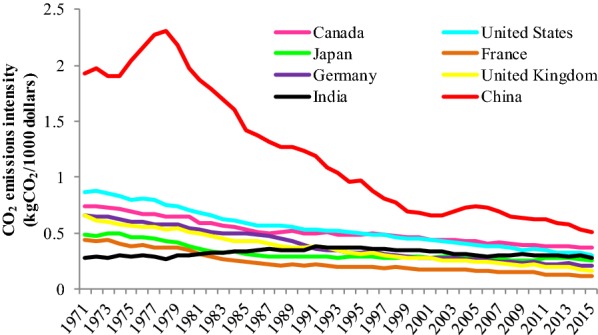
CO_2_ emissions intensity of the eight main countries between 1971 and 2015 (the main countries are eight historical or continual big emitters in the world. However, their carbon emissions intensity is improved gradually. Different color lines represent different countries’ carbon emissions intensity from fuel combustion between 1971 and 2015) Source: IEA 2017 [52]

Significant progress has been made in reducing the carbon emission intensity. The carbon emission intensity of China reduced rapidly as the total amount of carbon emissions was increased and higher than other countries since 2000. From 1971 to 2015, China’s carbon intensity was reduced substantially (73.9%), compared with the United States’ carbon intensity reduction (65.3%) (Fig. 3). Moreover, the Chinese government set more rigorous carbon emission intensity goals. For example, the government submitted its intended nationally determined contributions of enhancing actions on climate change to the UN in 2015. It declared that China would achieve peak carbon emissions around 2030 and make best efforts to peak early through reducing carbon emission per unit of GDP by 60–65% from the 2005 level.

**Fig. 4:**
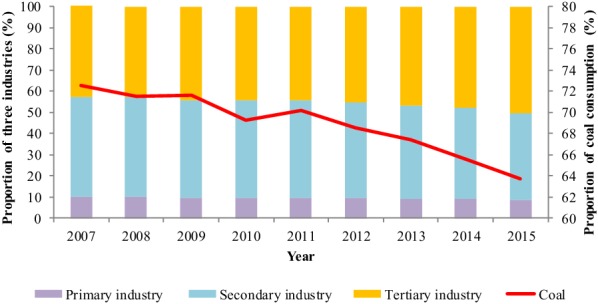
The proportion of all three industries and coal between 1971 and 2015 (different color rectangular represents the proportion of primary industry, secondary industry and tertiary industry respectively. The line represents the proportion of coal) Source: China statistical yearbook 2017 [36]

Policies issued by government have given an important institutional guarantee for energy saving, carbon emissions intensity reduction and industrial structure adjustment. Energy was saved by launching the Ten Key Energy Conservation Projects and the 1000 Enterprises Program. Remarkable achievements have been made to reduce carbon emissions intensity through eliminating backward production capacity [53]. Elimination of backward production capacity, commenced in 2007, mainly concentrating on energy intensity industries such as iron smelting, steel production, coke, paper, plate glass, aluminum production, and small thermal power generating units [54]. From 2007 to 2015, the two biggest physical production reductions in elimination of backward production capacity were cement and iron smelting at 102,700 and 21,089 Mt, respectively (Table 1). Eliminating backward production capacity has decoupled industrial output and fossil energy consumption intensity since 2015 [55]. For example, coal consumption in thermal power supply decreased from 370 to 333 g/kwh and comprehensive energy consumption per ton of steel decreased from 694 to 605 kg of standard coal with a decrease rate of 12.8% during 2005–2010 [34]. In addition, strategic emerging industries were proposed in 2010 so as to adjust energy and industrial structure gradually. We can learn that the proportion of coal consumption reduced from 72.5 to 63.7% during 2007–2015. The added value of service industry exceeded that of all industries’ in 2013. By 2015, the proportions of service and industry in GDP were 50.2% and 40.9%, respectively [36] (Fig. 4).

**Table 1:** Amount elimination of backward production capacity. Source: China’s Policies and Actions for Addressing Climate Change 2008–2016 [29, 32, 34, 41, 43, 44, 47, 56]

Time span	Iron smelting (Mt)	Steel production (Mt)	Cement (Mt)	Small thermal power generating unit (10^6^ kw)	Coke (Mt)	Paper (Mt)	Plate glass (10^6^ cases)	Electroly aluminum (Mt)
2007–2010	12,000	7200	37,000	7682	10,700	1130	4500	120.9
2011–2015	9089	9486	65,700	1732.8	4499	1887	16,900	205
Total	21,089	16,686	102,700	9414.8	15,199	3017	21,400	325.9

Carbon emission trading market construction has yielded positive results. The carbon emission trading market of China was launched later than that of developed countries; however, it developed rapidly, especially after 2015. Key-emission participators and the amount of carbon trading increased nearly thirteen- and two -fold, respectively. Accumulated trading quota of pilot carbon emissions trading market increased from 13.75 to 197 Mt CO_2_e, and the accumulated trading value increased from 0.5 to 45.52 billion between 2014 and 2017 (Table 2). At the end of 2015, there were seven cities and provinces launching carbon emission trading market pilots. The national carbon emission trading market of the electricity industry was launched in December 2017.

**Table 2:** The carbon emission trading market in China. Source: China’s Policies and Actions for Addressing Climate Change 2013–2017 [41, 43, 44]

Year	The number of involved sectors/key-emission enterprises	Accumulated trading	
		Money (billion RMB)	Quota (Mt CO_2_e)
2014	–/more than 1900	0.5	13.75
2015	20/more than 2600	2.3	67
2017	20/nearly 3000	45.52	197

Positive results have been achieved in circular economy and low carbon technologies. Firstly, China recycled 72 Mt of steel scrap, 5.2 Mt of nonferrous metals and more than 16 Mt of plastic [56] through the Circular Economy Pilots by 2009. Secondly, CCUS is a useful method of recycling carbon and increasing the carbon sink. Shenhua Group’s CCUS demonstration projects stored 57,000 tons of CO_2_ in total by 2012 and the first CO_2_ geological storage demonstration project in Ordos, Inner Mongolia sequestrated 12 Mt of CO_2_ by June 2013 [41]. Finally, a total of 2364 of CDM projects had been registered with the United Nations Clean Development Mechanism Executive Board by 2012, and accounted for 50.41% of the world’s total registered programs. Their estimated CER has reached 420 Mt of CO_2_e, accounting for 54.54% of the global total. Overall, China tops the list in both numbers of registered projects and annual CRE in the world (Table 3).

**Table 3:** Development of CDM projects in China. Sources: China’s Policies and Actions for Addressing Climate Change 2008–2012 [29, 32, 34, 47, 56]

Years	Cumulative registered projects with the UNCDMEB	Estimated CER (Mt CO_2_e)	Proportion of CER (%)
2008	244	113	–
2009	632	188	–
2010	1003	230	60.8
2011	1560	328	63.84
2012	2364	402	54.54

**Fig. 5:**
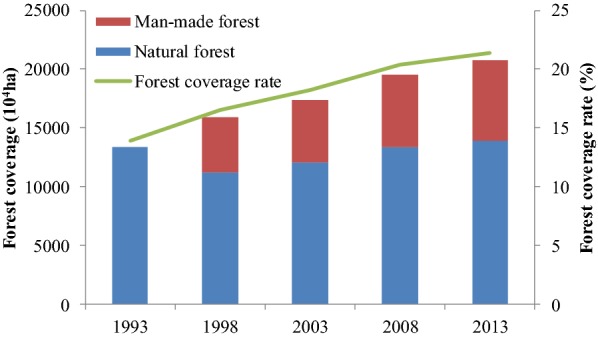
Annual forest coverage and its rate between 1993 and 2013 (red rectangular represents man-made forest and blue rectangular represents natural forest. Forest coverage contains both man-made and natural forestss) Source: Main Results of the Eighth National Inventory of Forest Resources (1993–2013) [57, 58]

Afforestation and agricultural carbon sink projects have greatly contributed to carbon emission reduction. According to Chinese national forest resource investigation, the area of man-made forest was continually increased and tops the world after 1998. The total annual forest coverage rate of China has increased vigorously from 13.92 to 21.36% between 1993 and 2013 [57, 58] (Fig. 5). And the AAGR of man-made forest coverage rate was 2.6%, higher than the total forest coverage rate’ AAGR (1.7%) in China over the period of 1993–2013. Vast forest strengthened the carbon sink function of terrestrial ecosystems in China. Eco-friendly Tilling and other agricultural projects have been conducted to increase the agricultural carbon sink. For instance, the total area of Eco-friendly Tilling reached around 8.60 million ha in 2014 [43]. Fertilizer projects were employed across total 60.00 million ha of cropland by 2008, which was estimated to reduce GHG emissions by 890 Mt CO_2_e [56].

The Chinese government has positively focused on the development of low carbon cities through energy-efficient buildings, green buildings, low-carbon city and sponge cities. Firstly, the area of energy-efficient buildings was 10.5 billion m^2^, accounting for 38% of the floor space in domestic cities and towns, equivalent to an annual saving of 0.1 billion tons coal equivalent and 260 Mt carbon emissions by 2014. Secondly, a total of 2.7 billion m^2^ area in cities and towns nationwide employed solar power, and 460 million m^2^ area used geothermal energy by 2014 [43]. Thirdly, 3979 projects nationwide have obtained green building labels, with a total construction area exceeding 450 million m^2^ by 2015 [44]. Finally, low-carbon pilot cities formulated their own implementation plan, which has helped establish an accountability scheme for meeting the GHG emission controlling target, strengthening the basic capacity for the GHG emissions accounting and inventory preparation, and promoting the establishment of low carbon industry, buildings, transportation and energy systems. The assessment of fulfillment of carbon intensity targets during the 12th FYP period showed that the carbon intensity in these pilot provinces and cities was much lower than that of the national average.

## Existing problems with China’s low carbon economy

As the most populated and the biggest developing country in the world, China faces many challenges in economic and social reform. Although China has made great achievements in low carbon development, some problems remain in the development of the low carbon economy such as the absence of top-level and long-term planning, local governments’ poor enthusiasm when carrying out central government’s regulations, and uniform policies lacking comprehensive consideration. The main problems are summarized as follows:

1. Many policies are made for short-term goals, which have led to policy overlap in China’s low carbon economy. Policies and actions in many pathways such as low carbon cities and carbon emission reduction tasks lacked long-term planning and even were repeated [59]. For example, China has launched several different ideas for low carbon city development, such as green city, sponge city, low carbon city and forest city. Those concepts lack long-term planning and were mainly stopgap measures [60], disturbing the development direction of cities. Furthermore, the carbon intensity reduction index was enacted to supervise the performance of local governments’ emission reduction target in the 12th FYP. Some provinces used extreme measures of power rationing and vehicle restrictions in winters to complete their political targets such as realizing the aims of the “blue-sky” days in a year. These were short-sighted policies mainly for political targets rather than environmental goals [26].
2. A national and complete carbon trading market that contains more industries is absent in China. The Chinese government enacted the national carbon emission trading market of the electricity industry in 2017. While, a national cap-and-trade system that contains more industries is absent currently in China. Furthermore, the carbon price is essential to the carbon market, and should consider the energy efficiency, technology, production characteristics of different industries. A unified and transparent carbon verification system is needed in the preparation of carbon quota allocation. In addition, China’s carbon allowance allocation methods were mainly learnt from the experience of the European Union (EU), which cannot be considered suitable for China. In general, there are many details (e.g., carbon verification methods, supervisory institutions and carbon allowance allocation) should be improved within the cap-and-trade system.
3. Local governments have less incentive than the central government when curbing carbon emissions in China. The central government set carbon emission reduction targets to encourage local government control regional carbon emission in China’s FYPs. However, local governments have more interest on GDP growth rather than carbon emissions reduction. For example, some local governments completed their carbon emissions intensity target by increasing GDP, through which both political economic goals and carbon emission intensity reduction targets will be realized simultaneously. However, inappropriate ways of expanding GDP led to increased carbon emissions. In addition, local governments predominantly chase economic prosperity despite the elimination of backward production. To maintain a GDP increase, some local governments protected carbon-intensive industries, on which their GDP boom relied. The most carbon-intensive industries are also typically the industries that possess backward production that central government have a strong desire to eliminate. Consequently, disharmony between central and local government has not only caused more carbon emissions but also slowed down the progress of eliminating the backward production.
4. Uniform policies are not suitable for different regions in China. China is a vast country with complicated natural and socioeconomic conditions that vary among different regions, especially the disparity between the rich and the poor. For example, China is promoting clean energy nationwide. For rich regions with mature technology and equipment, the employment of clean energy for carbon emission reduction will be easy. However, for the poor regions, clean energy usage will be difficult and most such areas still rely on traditional high carbon emission equipment. Therefore, uniform policies that envelop both rich and developing regions are not suitable. However, carbon-intense industries (e.g., electricity, cement, chemicals and processed metal), which generate considerable carbon emissions, are mainly developed in poor regions. Current carbon emission intensity assessment indexes are calculated based on the primary energy intensity [59]. As a result, some rich regions consumed the above products and services and completed the carbon emissions intensity targets easily, while leaving their embodied carbon and environmental burden to the poor regions.
5. China’s outdated production equipment, poor energy efficiency among small enterprises, and low secondary energy recovery rate are hindrance for energy saving, and may even lead to energy waste [61]. All the actions and policies are essentially aimed at improving energy efficiency. It is well accepted that many industrial technologies of China fall behind those of developed countries. The current low energy efficiency is mainly the result of underdeveloped technologies. Moreover, equipment substitution for advanced low carbon technology is also restricted by cost [62].
6. Public participation in carbon emission reduction less than governments’ in China. The government attaches great importance to energy conservation and carbon emission reduction and has thus made efforts to develop the low carbon economy. Thereby, many laws and regulations have been issued by governments to encourage the public to participate in the low carbon economy with an associated low carbon lifestyle. However, the public engagement is much weaker than the government’s expectation [40]. For example, waste of the water resource is very serious in daily life, even in some regions with severe water shortage because of the residents’ lack of water-saving consciousness. Garbage classification scheme is very difficult to carry out in most cities in China. Public electricity saving also remains weak because of the low interconnection between electricity price and energy price [63].

## Policy recommendations

Top-level planning should be involved in long-term low carbon development. For China’s low carbon cities, policy overlap was mainly caused by the crude theory of quantification and certification for low carbon cities despite the fact that low carbon cities are now appearing worldwide. Scientific and reasonable low carbon theories, such as a low carbon city indicator framework [23], are promising to provide top-level and long-term low carbon city planning for central government in the future. Moreover, consistency of low-carbon policies, long-term urban energy system planning [64] and industrial planning [65] are needed for developing low carbon cities, which will also contribute to the low-carbon development in the long run. The national carbon emission trading market and the mechanism for carbon verification, allocation and pricing also should be well designed at the top-level.

Rational assessment methods will be necessary not only for coordinating the relationship between central and local government but also for formulating fair carbon emission reduction policies among different regions. To coordinate the relationship between central and local government, the carbon emissions intensity should be assessed by physical output (e.g., per tones steel production), rather than GDP [66], thus avoiding reduction by increasing GDP. Moreover, poor regions have yielded carbon-intense production that was almost entirely consumed by rich regions, leading to considerable embodied carbon emissions. To narrow the gap between the rich and poor, embodied carbon emissions should be considered in assessment mechanism of carbon emissions intensity. Moreover, a carbon compensation mechanism should be established for different regions to balance unfair economic development and carbon emissions.

Low carbon technologies should be explored by which enterprises cooperate with scientific research institutions. Low-carbon technologies may temporal be expensive but strongly contribute to carbon emission reduction alongside cost minimization in the long term [67, 68]. Although some enterprises in China have made efforts to upgrade their outdated equipment and eliminate backward production capacity, the development of low carbon technology has been far from meeting future needs. Therefore, related enterprises and scientific research institutions should cooperate to explore the efficient low carbon technologies.

Public participation in the low carbon lifestyle should be further strengthened. Relevant laws and regulations are necessary to encourage public participation. Therefore, the government should strengthen low carbon propaganda to promote public participation in the future social and economic low carbon transition. From the perspective of embodied carbon emissions, resource conservation and recycling will strongly contribute to carbon emission reduction. Electricity price bidding is promising to raise public awareness of energy saving. Garbage classification scheme institutions should be formulated to encourage public engagement.

## Conclusions

As a responsible developing country, China has been devoted to reducing carbon emission intensity and has made a series of carbon emission targets simultaneously. Overall, China is making its pathways to a low carbon economy. Consequently, dramatic achievements have been made in different aspects of China’s low carbon economy. For example, carbon emission intensity has been reduced drastically. Industrial structure was adjusted accompanied by the optimization of the energy structure. A national unified carbon trading market has been established. However, some problems remain such as the weakness of public participation, the ineffectiveness of unified policies and the absence of long-term planning for low carbon development. In the future, top-level and long-term planning should be conducted for further low carbon economy development. Different carbon emissions intensity assessments and controlling policies should be applied in different regions to coordinate the relationship between central and local government as well as narrow the gap between the poor and rich regions. Enterprises should cooperate with scientific research institutions to explored low carbon technologies. Finally, relevant institutions should be regulated to realize a comprehensive low carbon transition in China through reasonable and feasible low carbon pathways. The low carbon transformation of China’s economy and society will not only benefit its own economic transition, but also greatly contribute to global climate change mitigation. Long-term planning for low carbon development will undoubtedly strengthen China’s position as a leader of global climate governance.

## Data Availability

The raw material, the analysis based on, are available in the National Bureau of Statistics (https://www.stats.gov.cn/tjsj/), National Forestry and Grassland Administration and National Park Administration (https://english.forestry.gov.cn/), national development and reform commission (https://www.ndrc.gov.cn/), National Development and Reform Commission (https://www.ndrc.gov.cn/).

## Abbreviations

UNFCCC: United Nations Framework Convention on Climate Change
OECD: Organization for Economic Co-operation and Development
GHG: Greenhouse Gas
EU-ETS: European Union’s Emissions Trading Scheme
CDM: Clean Development Mechanism
FYP: Five-year Plan
MIIT: Ministry of Industry and Information Technology
NDRC: National Development and Reform Commission
AAGR: Average Annual Growth Rate
CCUS: Carbon Capture, Use and Storage
CER: Certified Emissions Reduction
MST: Ministry of Science and Technology
REDD: Reduced Emissions from Deforestation and Forest Degradation
MOA: Ministry of Agriculture
EU: European Union
IEA: International Energy Agency
UNCDMEB: United Nations Clean Development Mechanism Executive Board
